# Trends in Medicare Reimbursement for Interventional Radiology Procedures: 2007–2020

**DOI:** 10.7759/cureus.43840

**Published:** 2023-08-21

**Authors:** Soryan Kumar, Aditya Khurana, Jack Haglin, Rohit Khurana, Douglas Hidlay, Adam E. M Eltorai

**Affiliations:** 1 Medical School, The Warren Alpert Medical School of Brown University, Providence, USA; 2 Radiology, Mayo Clinic, Rochester, USA; 3 Orthopedic Surgery, Mayo Clinic, Phoenix, USA; 4 Molecular and Cellular Biology, Vanderbilt University, Nashville, USA; 5 Radiology, Augusta Health, Fisherville, USA; 6 Radiology, Brigham and Women's Hospital, Boston, USA

**Keywords:** trend analysis, insurance reimbursement, centers for medicare and medicaid services, health policy and economics, interventional radiology

## Abstract

Purpose: Declining physician reimbursement has been occurring across multiple specialties due to changes in Medicare legislation, including the Deficit Reduction Omnibus Reconciliation Act (DRA), the Balanced Budget Act, and the Sustainable Growth Rate. The purpose of this study was to evaluate trends in Medicare reimbursement rates for various procedural classes in interventional radiology from 2007 to 2020.

Methods: Common interventional radiology procedures were selected across multiple procedural classes: gastrointestinal, biliary, urinary, fallopian dilatation, other injection/change/removal, iliac vascular, femoral/popliteal vascular, tibial/peroneal vascular, hepatobiliary, and vascular emergency. The Physician Fee Schedule Look-Up Tool from the Centers for Medicare & Medicaid Services was queried for current procedural terminology (CPT) codes to extract reimbursement data. All monetary data were adjusted for inflation using the United States consumer price index (CPI). The compound annual growth rate (CAGR) and average annual change in reimbursement were calculated based on these adjusted trends.

Results: Aside from urinary and vascular emergency procedures, all other procedural classes experienced decreases in inflation-adjusted Medicare reimbursement from 2007 to 2020. The greatest mean decrease in reimbursement rates was observed in biliary procedures (-$21.25), while the largest mean increase in reimbursement rates was observed in vascular emergency procedures ($3.23). All procedures with increases in reimbursement rates and 36.8% of procedures with decreases in reimbursement rates have a CPT code change between 2007 and 2020.

Conclusion: After accounting for inflation, reimbursement rates were shown to decline for all procedural classes except for urinary and vascular emergencies. Congressional policies, such as the Deficit Reduction Act (DRA) and the Medicare Access and Children's Health Insurance Program (CHIP) Reauthorization Act of 2015, may clarify some of these trends.

## Introduction

Understanding physician reimbursement is critical for practice sustainability. Declining physician reimbursement has been a generalized phenomenon across multiple specialties due to recent changes in Medicare legislation, such as the Deficit Reduction Omnibus Reconciliation Act of 2005 (DRA) [1], the establishment of the Balanced Budget Act (BBA) [2], and the creation of the Sustainable Growth Rate (SGR) [3] in particular. Despite the importance and impact of these cuts to healthcare spending, there has been little research to evaluate the Medicare reimbursement trends for interventional radiology (IR) over this period. IR is a new field relative to most medical specialties and has seen significant development in its clinical practice [4]; however, rising healthcare costs may present new challenges to this growing specialty. As no significant changes in IR procedural complexity and physician effort have been noted, any observed trends may be attributed to changes in Medicare legislation.

Effectively modeling and understanding of these trends is a critical aspect of sustaining IR practices across the United States (US) going forward. Given the lack of a comprehensive review of reimbursement trends in IR, this study intends to describe recent national Medicare reimbursement trends for IR.

## Materials and methods

Healthcare billing in the US centers on the use of Current Procedural Terminology codes (CPT) to describe and categorize the breadth of reimbursed medical procedures. A resource-based relative value scales all resource costs into consideration to calculate the appropriate reimbursement rate for each procedure [5,6]. Furthermore, Medicare multiplies each reimbursement rate by a geographic practice cost index to account for cost variation across geographic regions. Centers for Medicare & Medicaid Services (CMS) updates each reimbursement rate per CPT code annually, factoring in changes to input resource costs [7].

This study includes common procedures detailed by Chand et al. in *Essential Interventional Radiology Review* under the following 10 procedural classes: gastrointestinal, biliary, urinary, fallopian dilatation, other injection/change/removal, iliac vascular, femoral/popliteal vascular, tibial/peroneal vascular, hepatobiliary, and vascular emergency [8]. For each of the selected procedures, the Physician Fee Schedule Look-Up Tool from the CMS was queried to obtain each procedural CPT code and to track any CPT code changes from 2007 to 2020. Medicare reports reimbursement for each procedure through its respective CPT code for a given year. Reimbursement data was collected as national payments under the global modifier. Subsequently, all monetary data were adjusted to 2020-dollar values to compare reimbursement trends over time across each of the procedural classes using the latest consumer price index data from the US Department of Labor and Bureau of Labor Statistics [9]. The Physician Fee Schedule lacked reimbursement data for certain procedures during some years due to either the absence of a CPT code or uncollected data for certain codes. Any procedures with more than four years of missing data from the Physician Fee Schedule Look-Up Tool were excluded since data were consistently unavailable between 2007 and 2011 for these procedures.

For each procedure, total percentage changes were calculated from 2007 to 2020. Compound annual growth rates (CAGRs) were also computed to provide a simplified yearly growth rate for procedures from 2007 to 2020 by reducing the inherent data fluctuation. Each CAGR was determined with adjusted data using the following formula:

$CAGR = \left[ \left( \frac{2020 ~ Value}{2007 ~ Value} \right)^{1/(2020-2007)} \right] - 1$

For any procedure missing data in 2007, the next available year was substituted in the CAGR formula. Overall differences in yearly reimbursements by procedural class were approximated through a least-squares regression line of reimbursement rates by year. Mean annual change is given as the slope of this regression. Mean unadjusted total percent change was calculated without adjusting for inflation. All other depicted reimbursement figures, percentage changes, and growth rates are adjusted for inflation. Statistical significance is assessed via linear regression t-test and evaluated at α = 0.05.

## Results

A total of 79 procedures were provided under the above 10 procedural classes of which 10 (12.6%) were excluded given the above exclusion criteria. Out of the remaining 69 IR procedures presented in this analysis, 33 (47.8%) experienced a CPT code change between 2007 and 2020. These CPT code changes are detailed in Table 1. Of these 33 code changes, 29 resulted from code branching, while the remaining four correspond to a code replacement. All procedures under iliac vascular, femoral/popliteal vascular, and tibial/peroneal vascular were missing data from 2007-2010, so results for these classes were computed over 2011-2020.

**Table 1:** Selected Interventional Radiology Procedures G-Tube: gastrostomy tube; J-Tube: Jejunostomy tube; PTA: percutaneous transluminal angioplasty; TIPS: transhepatic intrajugular portosystemic shunt; HSG: hysterosalpingogram; GJ Tube: gastrostomy-jejunostomy tube; CPT: Current Procedural Terminology

Procedural Class	CPT Code(s)	Description	Code Changes	Missing Years
Gastrointestinal	43750/49440	G-Tube Placement, Percutaneous	Branching code	
	43750/49441	J-Tube Placement, Percutaneous	Branching code	
	43750/49442	Cecostomy or other Colonic Tube	Branching code	
	49446	Convert G-Tube to G-J Tube		2007
	43760/49450	G-Tube Change (with Fluoroscopy)	Branching code	
	43760/49451	Replacement Dislodged J-Tube	Branching code	
	43760/49452	Replacement GJ Tube, Percutaneous	Branching code	
	43760/43762	Replacement GJ Tube, Percutaneous including remove without imaging/without revision tract	Branching code	
	43760/43763	Replacement GJ Tube, Percutaneous including remove without imaging/without revision tract	Branching code	
	43761	Repositioning of Nasogastric/Orogastric FeedingTube		
	49465	Contrast Injection for any GITube		2007
	43453	Esophageal Dilation Over Guide Wire		
	43752	Nasogastric Tube Placement		
	49460	Mechanical Removal Obstructed Material		2007
	49400	Injection of Air or Contrast into Peritoneal Cavity		
Biliary	47490	Percutaneous Cholecystostomy		
	47500/47531	Injection for Cholangiogram, Existing access	Replacement code	
	47505/47532	Injection for Cholangiogram, New access	Replacement code	
	47510/47533	Percutaneous Placement Billiary Drainage (Ext.)	Branching code	
	47510/47534	Percutaneous Plcmt Billiary Drainage (Int.-Ext.)	Branching code	
	47530/47535	Convert External Biliary Drainage to Internal-External	Branching code	
	47525/47536	Exchange of Biliary Drainage Catheter	Branching code	
	47530/47537	Removal of Biliary Drainage Catheter	Branching code	
	47511/47538	Placement Bile Duct Stent(s), Existing access	Branching code	
	47511/47539	Placement Bile Duct Stent(s), New without drainage	Branching code	
	47511/47540	Placement Bile Duct Stent(s), New with drainage	Branching code	
	47541	Placement Access Through Biliary Tree, Percutaneous, new access		2007-2015 (Excluded)
	47542	Balloon Dilation Biliary Duct/Ampulla, Percutaneous		2007-2015 (Excluded)
	47543	Endoluminal Biopsy of Biliary Tree, Percutaneous		2007-2015 (Excluded)
	47544	Removal calculi/debris from Bile Duct(s)/Gall Bladder		
Urinary	50390	Aspiration/injection of Renal Cyst or Pelvis, Percutaneous		
	50395/50436	Dilation of Existing Tract, Percutaneous, including imaging guidance	Branching code	
	50395/50437	Dilation of Exist Tract, Percutaneous, including imaging guidance; new access	Branching code	
	50394/50430	Injection Antegrade NephroUureterogram, New access	Branching code	
	50394/50431	Injection Antegrade Nephro/Ureterogram, Existing access	Branching code	
	50392/50432	Placement Nephrostomy Catheter, Percutaneous including diagnostic nephrogram	Branching code	
	50392/50433	Placement Nephroureteral Catheter, Percutaneous including diagnostic nephrogram	Branching code	
	50392/50434	Convert Nephrostomy Catheter	Branching code	
	50398/50435	Exchange Nephrostomy Catheter	Replacement code	
	50387	Remove and Replace External Nephroureteral Catheter		
	50606	Endoluminal Biopsy of Ureter/Renal Pelvis		2007-2015 (Excluded)
	50393/50693	Placement of Ureteral Stent, Existing nephrostomy tract	Branching code	
	50393/50694	Placement of Ureteral Stent, New access without separate nephrostomy catheter	Branching code	
	50393/50695	Placement of Ureteral Stent, New access with separate nephrostomy catheter	Branching code	
	50705	Ureteral Embolization or Occlusion		2007-2015 (Excluded)
	53854	Transurethral Destruction of Prostate, By radiofrequency		2007-2015 (Excluded)
	50706	Balloon Dilation, Ureteral stricture		2007-2015 (Excluded)
Fallopian	58340	HSG		
	58345	Fallopian Dilatation		
Other Injection, Change, Removal	50688	Change of Ureterostomy Tube/Ureteral Stent		
	50396	Whitaker Test		
	50080	Nephrostolithotomy <2cm		
	50081	Nephrostolithotomy >2cm		
	50690	Ileoconduit Injection		
	51600	Injection Cystogram/Voiding Urethrocystogram		
	51605	Cystography/Voiding cystourethrography with chain		
	51610	Urethrocystogram, Retrograde		
	51705	Change Cystostomy Tube, Simple		
	51710	Change Cystostomy Tube, Complex		
Iliac	37220	PTA, Unilateral		2007-2010
	37221	Stent Placement(s) with PTA When Performed, Unilateral		2007-2010
	37222	PTA Each Additional Ipsilateral Iliac Vessel		2007-2010
	37223	Stent Placement(s) with PTA Within Same Vessel When Performed, Each additional vessel		2007-2010
Femoral	37224	PTA, Unilateral		2007-2010
	37225	Atherectomy with PTA Within Same Vessel When Performed, Unilateral		2007-2010
	37226	Stent Placement(s) with PTA Within Same Vessel When Performed, Unilateral		2007-2010
	37227	Stent and Atherectomy with PTA Within Same Vessel When Performed, Unilateral		2007-2010
Tibial	37228	PTA, Unilateral		2007-2010
	37229	Atherectomy with PTA Within Same Vessel When Performed, Unilateral		2007-2010
	37230	Stent Placement(s) with PTA Within Same Vessel When Performed, Unilateral		2007-2010
	37231	Stent and Atherectomy with PTA Within Same Vessel When Performed, Unilateral		2007-2010
Hepatobiliary	37182	TIPS		
	36470	Sclerotherapy in a single vein		
	50684	Injection for Ureter X-ray		
	37210/37243	Vascular Embolization/Occlude Organ	Branching code	
	37204/37242	Vascular Embolization/Occlude Artery	Branching code	
Vascular Emergency	61626	Transcatheter Permanent Occlusion or Embolization		
	37204/37244	Vascular Embolization/Occlude Bleed	Branching code	
	34900/34708	Endovascular Repair of Infrarenal/Iliac Arteries	Branching code	

**Figure 1:**
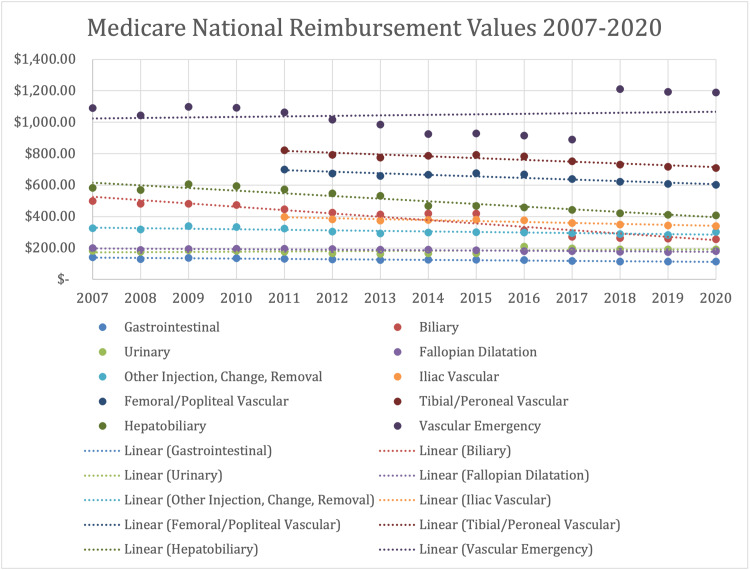
Mean Adjusted Medicare National Reimbursement Rates by Procedural Class from 2007-2020 Iliac Vascular, Femoral/Popliteal Vascular, and Tibial/Peroneal Vascular procedural classes are shown over 2011-2020 due to missing reimbursement data.

Prior to adjusting for inflation, five out of the 10 aggregate procedural groups experienced average reimbursement rate increases, including GI, urinary, fallopian dilatation, other injection/change/removal, and vascular emergency. After adjusting for inflation, urinary and vascular emergency procedures experienced average annual reimbursement rate increases without statistical significance. The remaining eight groups depict average annual reimbursement rate decreases with statistical significance at α = 0.05. Vascular emergency procedures had the largest annual increase ($3.23) with a 9.1% increase from 2007 to 2020, while biliary procedures had the largest annual decrease (-$21.25) with a 48.7% decrease from 2007 to 2020. Average reimbursement trends for the iliac vascular, femoral/popliteal vascular, and tibial/peroneal vascular procedure classes are presented in Table 2 from 2011 to 2020, while all other procedure classes are presented in Table 2 from 2007 to 2020. Average reimbursement fee schedules for each procedural class are presented in Table 3 and Figure 1.

**Table 2:** Mean Adjusted Reimbursement Trends from 2007 to 2020 Iliac Vascular, Femoral/Popliteal Vascular, and Tibial/Peroneal Vascular procedural classes are computed over 2011-2020 due to missing reimbursement data. CAGR: Compound annual growth rate

Procedural Class	Mean CAGR	Mean Annual Change	Mean Unadjusted Total Percent Change	Mean Total Percent Change	P-Value
Gastrointestinal	-1.7%	-$2.02	-0.7%	-20.1%	<0.0001
Biliary	-5.0%	-$21.25	-36.2%	-48.7%	<0.0001
Urinary	0.3%	$1.37	30.1%	4.6%	0.1412
Fallopian Dilatation	-0.8%	-$1.70	-10.1%	11.8%	<0.0001
Other Injection, Change, Removal	-0.5%	-$3.36	-6.6%	16.0%	0.0005
Iliac Vascular	-1.7%	-$5.96	-14.7%	-2.2%	<0.0001
Femoral/Popliteal Vascular	-1.7%	-$9.85	-14.0%	-1.4%	0.0001
Tibial/Peroneal Vascular	-1.6%	-$11.42	-13.8%	-1.2%	<0.0001
Hepatobiliary	-2.7%	-$16.78	-30.2%	-13.2%	<0.0001
Vascular Emergency	0.7%	$3.23	9.1%	35.6%	0.6691

**Table 3:** Mean Adjusted Reimbursement Fee Schedule by Procedural Class from 2007-2020 Iliac Vascular, Femoral/Popliteal Vascular, and Tibial/Peroneal Vascular procedural classes are computed over 2011-2020 due to missing reimbursement data.

Procedural Class	2007	2008	2009	2010	2011	2012	2013	2014	2015	2016	2017	2018	2019	2020
Gastrointestinal	$ 141.40	$ 129.96	$ 137.08	$ 135.22	$ 131.40	$ 126.32	$ 123.56	$ 125.90	$ 126.04	$ 123.67	$ 117.04	$ 114.07	$ 113.73	$ 112.94
Biliary	$ 498.94	$ 481.20	$ 482.18	$ 474.11	$ 445.44	$ 424.42	$ 413.55	$ 419.53	$ 418.80	$ 312.05	$ 271.05	$ 263.92	$ 258.77	$ 256.03
Urinary	$ 182.12	$ 176.37	$ 185.98	$ 181.67	$ 174.97	$ 166.86	$ 162.18	$ 166.00	$ 166.17	$ 209.69	$ 197.05	$ 192.28	$ 192.28	$ 190.58
Fallopian Dilatation	$ 199.03	$ 188.29	$ 192.70	$ 195.74	$ 195.46	$ 192.45	$ 189.82	$ 189.99	$ 186.34	$ 180.16	$ 180.28	$ 175.62	$ 174.01	$ 179.01
Other Injection, Change, Removal	$ 324.71	$ 317.44	$ 339.36	$ 333.40	$ 322.75	$ 304.38	$ 292.87	$ 298.59	$ 301.17	$ 297.86	$ 293.66	$ 288.38	$ 282.66	$ 303.12
Iliac Vascular	-	-	-	-	$ 398.23	$ 382.72	$ 373.81	$ 377.34	$ 380.16	$ 375.60	$ 359.62	$ 349.94	$ 342.66	$ 339.78
Femoral/Popliteal Vascular	-	-	-	-	$ 699.80	$ 673.61	$ 659.11	$ 666.02	$ 675.13	$ 667.46	$ 637.87	$ 620.84	$ 607.58	$ 602.07
Tibial/Peroneal Vascular	-	-	-	-	$ 822.54	$ 791.62	$ 775.68	$ 786.56	$ 792.03	$ 782.92	$ 750.95	$ 730.93	$ 716.74	$ 709.25
Hepatobiliary	$ 583.27	$ 567.96	$ 606.24	$ 594.65	$ 572.30	$ 547.60	$ 531.73	$ 468.29	$ 466.81	$ 458.37	$ 442.34	$ 420.00	$ 410.79	$ 407.38
Vascular Emergency	$ 1,089.26	$ 1,044.01	$ 1,097.82	$ 1,092.29	$ 1,061.94	$ 1,015.36	$ 984.02	$ 923.91	$ 928.44	$ 915.59	$ 889.76	$ 1,209.83	$ 1,193.48	$ 1,188.31

Out of the 69 examined procedures, 12 (17.4%) resulted in an overall reimbursement increase, although all of these procedures experienced a CPT code change between 2007 and 2020. Of the remaining 57 procedures with overall reimbursement decreases, 21 (36.8%) procedures experienced a CPT code change between 2007 and 2020. Over this time period, endovascular repair of infrarenal aorta (under vascular emergency procedures) had the largest increasing annual reimbursement rate ($58.06), while placement of bile duct stent for existing access (under biliary procedures) had the largest decreasing rate (-$43.78). Both of these procedures had branching CPT codes in addition to other procedures with large shifts in annual reimbursements as shown in Tables 1, 2. Biliary procedures encompass many of the individual procedures with large annual decreases, while urinary procedures contained eight (33.3%) of all increasing procedures.

## Discussion

This study analyzes Medicare reimbursement trends from 2007 to 2020 for IR procedures over 10 procedural classes: gastrointestinal, biliary, urinary, fallopian dilatation, other injection/change/removal, iliac vascular, femoral/popliteal vascular, tibial/peroneal vascular, hepatobiliary, and vascular emergency. The results indicate a statistically significant decrease for all procedural groups except urinary and vascular emergency procedures after adjusting for inflation. All procedures with average increases in reimbursements were noted to have a CPT code change between 2007 and 2020. As CPT code replacements may not be perfect due to code branching, procedures with code changes were typically noted to have sudden changes in annual reimbursements at the year of the code change.

Although a previously published article has analyzed Medicare reimbursement trends for interventional radiology through an analysis of the top 20 procedures from 2012-2020, our work provides a more in-depth analysis into trends for specific procedural groups from 2007-2020 and encompasses data collection on 79 procedures [10]. This work complements our analysis nicely as it sets the stage for the slow decline in reimbursement trends within IR.

Although the scope of IR has expanded, reimbursements to physicians from Medicare are decreasing [11]. Congressional policies between 2007 and 2020 may clarify some, though not all, of these trends. The enactment of the SGR, as part of the Balanced Budget Act of 1997, focused on significant reductions in Medicare reimbursements to physicians and hospitals. The SGR remained active as an annual cost-saving measure until its repeal in 2015. If healthcare costs were to exceed a calculated growth rate, the SGR would automatically truncate reimbursement the following year to match. Similar trends are evident in diagnostic radiology [12], gastroenterology [13], and orthopedic surgery [14], according to the previous literature. Declining reimbursements were even more pronounced, starting in 2007, when reductions per the DRA went into effect. This continued until the SGR was finally annulled through the passage of the Medicare Access and CHIP Reauthorization Act of 2015 (MACRA). In place of the SGR, MACRA prescribed a fixed 0.5% fee increase per year from 2015 to 2019. By 2020, the planned reimbursement increases are scheduled to halt, and it remains to be seen how these policies will affect Medicare reimbursement within IR in the future [15-17].

Beyond 2020, CMS has issued and adopted the Medicare Physician Fee Schedule Final Rule for Calendar Year 2021. The final rule, which went into effect on January 1, 2021, includes streamlined reporting and documentation processes, as well as increased reimbursement allocated for outpatient evaluation and management (E/M) services. However, these changes occurred within the confines of Medicare’s budget neutrality, which requires that increases in value for given services must be offset by equivalent decreases in payments for other services. Therefore, under budget neutrality, the increases assigned to E/M services prompted a large decrease in the 2021 conversion factor for determining Medicare reimbursement for physicians, as well as additional cuts to many services across medicine.

This decrease disproportionately affected radiology, as the implemented Medicare Fee Schedule for 2021 included an 11% cut to reimbursement within radiology as a whole, including a 9% cut to IR [18]. The Society of Interventional Radiology has released a public comment on the fee schedule in an effort to reverse these cuts [19]; however, this has been unsuccessful at the time of this writing. However, Congress passed the Consolidated Appropriations Act, 2021 (H.R. 133), which increases Medicare payments for all services furnished by physicians in the year 2021 by 3.75% in order to address the challenges presented by coronavirus disease 2019 (COVID-19) [20]. Although this temporarily may help alleviate some of the impacts of the cuts to radiologists, it remains to be seen how the ongoing changes being discussed and implemented by CMS will affect reimbursement within IR moving forward.

Limitations

Several of the procedures included in this study lacked reimbursement data for earlier years due to CPT code changes or the absence of a CPT code. With the potential of these procedures to severely skew their procedural class, incorporating them would have led to major inconsistencies in the observed mean values. In general, procedures with four or more missing years were not used for this analysis. Certain procedures with CPT code changes have also depicted large shifts in reimbursement rates. Particularly, many of the procedures under the urinary procedural class with average reimbursement increases, such as 50430 and 50695, were noted to have large reimbursement increases at the year of the code change. Likewise, some of the procedures under the biliary procedural class with large decreases, such as 47537 and 47544, were noted to have sudden drops in reimbursement rates at the year of the code change. Additionally, some entire classes of codes-iliac, femoral, and tibial-did not have CPT codes until 2011. The reimbursement trends for these codes were calculated but were presented separately in the corresponding tables. These categories also exhibited decreases in reimbursement, but it is important to note that these values cannot be compared directly to the analysis starting from 2007.

## Conclusions

This study currently offers a holistic review of Medicare reimbursement in interventional radiology from 2007 to 2020 and suggests that IR advocacy on a national level will be critical going forward given the impact that Congressional policy has had. Despite its public availability and standardization, the sole use of Medicare reimbursement data may not strictly reflect private insurance reimbursement trends. However, this study remains a practical surrogate of general reimbursement patterns since CMS decisions tend to influence the market as a whole. Further investigation is warranted to provide the foundation necessary to combat declining rates and sustain IR practices nationally as reimbursement decreases in specific procedural groups may impact the procedures that practices choose to conduct.

Decreasing Medicare reimbursements across most procedural classes indicates a necessity for engagement in current and future congressional policy. Advocacy at both the individual physician and professional organization level may be warranted to address decreases in any given procedural group. While physician and policymaker acknowledgment and understanding of these results are vital, their involvement in creating a sustainable reimbursement model to guarantee continued patient access to IR services must be an even higher priority.
